# Economy of scale for green hydrogen-derived fuel production in Nepal

**DOI:** 10.3389/fchem.2024.1347255

**Published:** 2024-04-08

**Authors:** Biraj Singh Thapa, Bishnu Pandey, Rahul Ghimire

**Affiliations:** Green Hydrogen Laboratory, School of Engineering, Department of Mechanical Engineering, Kathmandu University, Dhulikhel, Nepal

**Keywords:** synthetic natural gas, techno-economic analysis, economy of scale, green hydrogen, CO_2_ utilization

## Abstract

Opportunity for future green hydrogen development in Nepal comes with end-use infrastructural challenges. The heavy reliance of industries on fossil fuels (63.4%) despite the abundance of hydroelectricity poses an additional challenge to the green transition of Nepal. The presented work aims to study the possibility of storing and utilizing spilled hydroelectricity due to runoff rivers as a compatible alternative to imported petroleum fuels. This is achieved by converting green hydrogen from water electrolysis and carbon dioxide from carbon capture of hard-to-abate industries into synthetic methane for heating applications via the Sabatier process. An economy-of-scale study was conducted to identify the optimal scale for the reference case (Industries in Makwanpur District Nepal) for establishing the Synthetic Natural Gas (SNG) production industry. The techno-economic assessment was carried out for pilot scale and reference scale production unit individually. Uncertainty and sensitivity analyses were performed to study the project profitability and the sensitivity of the parameters influencing the feasibility of the production plant. The reference scale for the production of Synthetic Natural Gas was determined to be 40 Tons Per Day (TPD), with a total capital investment of around 72.15 Million USD. Electricity was identified as the most sensitive parameter affecting the levelized cost of production (LCOP). The 40 TPD plant was found to be price competitive to LPG when electricity price is subsidized below 3.55 NPR/unit (2.7 c/unit) from 12 NPR/unit (9.2 c/unit). In the case of the 2 TPD plant, for it to be profitable, the price of electricity must be subsidized to well below 2 NPR/kWh. The study concludes that the possibility of SNG production in Nepal is profitable and price-competitive at large scales and at the same time limited by the low round efficiency due to conversion losses. Additionally, it was observed that highly favorable conditions driven by government policies would be required for the pilot-scale SNG project to be feasible.

## 1 Introduction

Despite the positive advancements in renewable energy, such as increased solar PV installations and electric car sales, a new record for global carbon dioxide (CO_2_) emissions was set, with 37 billion metric tons (Gt) in 2022. The amount was 1% higher than previously anticipated (Grubler et al., 2018; Hussain et al., 2021; International Energy Agency, 2023). According to a study, there is an emphasis on the necessity for global CO_2_ emissions to be restricted by approximately 45% from 2010 levels by 2030, with the goal of reaching net zero by 2050 (Leahy et al., 2020; Intergovernmental Panel on Climate Change, 2022). To attain this, an urgent need exists for energy sources that are both sustainable, scalable, and adaptable, offering high energy density as viable alternatives to ensure a secure energy supply while mitigating the environmental impact associated with current non-renewable energy sources (Li et al., 2017). As a promising, low or zero-carbon energy source, hydrogen is acknowledged with significant potential as an energy carrier in the future. Projections suggest that green hydrogen will have a pivotal role in the global energy transformation (International Energy Agency, 2015)., given its adaptability and high heat value (120–140 MJ/kg) compared to gasoline (44 MJ/kg) and coal (20 MJ/kg) (Hosseini and Wahid, 2016; Francesco, 2018). Additionally, its use in CO_2_ recycling via the Sabatier Process offers energy-efficient solutions for addressing global energy demand and combating global warming (Dutta, 2014; Shastri et al., 2022).

**FIGURE 1:**
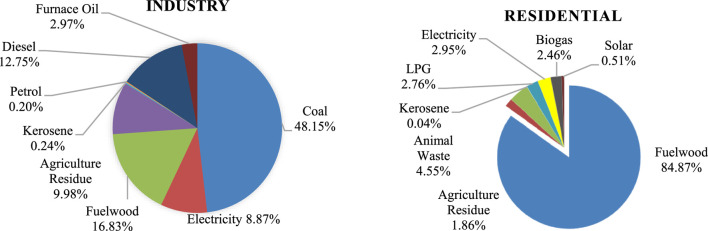
Energy mix of Nepalese Industrial and Residential Sector (Water and Energy Commission Secretariat, 2022).

Nepal’s low greenhouse gas emissions, coupled with its vulnerability due to melting Himalayas, drive the search for carbon-neutral solutions. However, the country’s annual investment of nearly 10% of its GDP, approximately 200 million USD, in fossil fuel imports is expected to rise due to population growth, inefficient operation, and increased economic production (Bhandari and Pandit, 2018; Nepal Oil Corporation, 2023). In Nepal, the primary energy source for 84.87% of households is fuel wood, while Liquefied Petroleum Gas (LPG) has experienced a 2.76% increase in usage over the last decade with over 33.1% of households employing it, particularly in urban areas, where it constitutes the second most common cooking fuel at 54.1% (Figure 1) (Water and Energy Commission Secretariat, 2022). Similarly, fossil fuels are heavily relied upon by Nepalese industries with a total energy consumption of 114.5PJ (Water and Energy Commission Secretariat, 2022) which is predominantly coal (48%) worth NRS 27.19 billion, imported in the fiscal year 2020–21, according to Nepal Rastra Bank.

On the other hand, Nepal has abundant renewable energy in the form of hydropower and solar resources with a current generation capacity of 2684 MW of hydroelectricity in 2023. (Nepal Electricity Authority, 2023). Nepal has 7231.3 MW hydropower projects under construction, with 20,000 MW in development stages. By 2030, the 16,820 GWh of surplus energy is projected. Exporting excess hydroelectricity faces geopolitical and pricing hurdles. Developing high-energy industries and grid management is a rapid government and developer task (Thapa et al., 2021). Moreover, over 90% of Nepal’s existing hydropower plants are runoff river type (Bhatt 2017) poses problems related to seasonal peaking, and in the absence of a lack of energy storage facilities Green Hydrogen and Hydrogen synthetic fuels provide better energy management opportunities.

Surplus hydropower can make 67,277 to 336,400 tons of green hydrogen with the use of 20% and 100% surplus energy in 2030, convertible to Synthetic Natural Gas using CO_2_ from cement, addressing compatible energy needs (Warsi et al., 2020; Thapa et al., 2021). Given the inadequacy of current hydrogen infrastructure and imbalance in electricity supply during dry seasons, the conversion to Synthetic Natural Gas (SNG) is deemed necessary. Annually, 3.6 million MT of CO_2_ is emitted by 72 cement plants in Nepal, with the potential for 1.3 million MT of SNG production as a substitute for heating fuel. (Zimmermann et al., 2020; Szima and Cormos, 2021). According to the Power-to-Gas concept, SNG can be produced by hydrogenating CO_2_ as following chemical equation:

$${\text{CO}}_{2}+4H_{2}\overset{\text{Ru}/\gamma -{\text{Al}}_{2}O_{3}/365\;℃}{\to }{\text{CH}}_{4}+2H_{2}O\;\Delta H_{R}\left(298\;K\right)=-165\text{ KJ}/\text{mol}$$ (1)

Methanation, a commonly employed technique for carbon monoxide and carbon dioxide elimination in chemical processes like ammonia production and natural gas purification, typically involves the conversion of small amounts of carbon dioxide. Bulk conversion can be hindered by the potential for numerous side reactions. Despite this, 128 Power-to-Gas (PtG) projects are recognized in Europe, with 27 already completed and 38 scheduled for future commissioning (Wulf et al., 2018). The CO_2_ methanation plants are primarily of small scale, while the 6 MW Audi e-gas plant is an exception. Electrolysis gained prominence when, in 2014, it was coupled with a methanation reactor and a coal-powered plant by Buchholz et al., 2014, achieving 53% efficiency, later theoretically claimed to be improved up to 80% for solid oxide electrolysis cell by Giglio et al., 2015, which has not reached technological maturity yet. The cost of hydrogen is a significant factor in SNG production, as identified by Szima and Cormos, 2021. A techno-economic assessment for SNG production was carried out by Becker et al., 2019, including the Organic Rankine Cycle for heat recovery, reporting SNG production costs as low as ∼2 USD/kg.

In Nepal, the concept of hydrogen and Synthetic Natural Gas is relatively new, and no work has been done to find the feasibility of the SNG plant. The study focuses on the process design, economy of scale, and a techno-economic assessment for the reference and pilot scale SNG plants focusing on the Hetauda industrial area in Makwanpur District.

## 2 Methodology

The present investigation consists of the development of a simulation model for SNG production utilizing ASPEN Plus Software to find out the required sizing of the equipment. The manufacturer’s data and economic analysis tools were used to analyze the economics of SNG production for the sized equipment of different scales. The risk analysis was done using Monte Carlo simulations. The following section details the requisite input, distinct process simulation models, and the resulting output.

### 2.1 Resources

In Nepalese industries, despite the bad reliability the older technologies for thermal purposes were gradually being replaced by electricity, coal and fuelwood continued to dominate. During COVID-19 restrictions, a 5.8% decrease in energy consumption was experienced in the sector in 2020, but an impressive 29% growth was witnessed in 2021, demonstrating the sector’s adaptability and recovery capabilities (Water and Energy Commission Secretariat, 2022).

The Hetauda Industrial District is one of Nepal’s largest industrial hubs located in Makwanpur District, encompassing 103 industries across various sectors, including plastic, cement, and mining industries. In this district, the industrial sector accounts for 813,800 GJ of energy consumption which is mostly carbon-emitting fuel (72%) (Makwanpur District District Development Committee Makwanpur Government of Nepal Ministry of Environment Alternative Energy Promotion Centre, 2011). The study is conducted to address the unreliable nature of hydroelectricity in Nepal, particularly stemming from runoff river projects. It aims to explore the conversion to e-fuel, which can be adapted and utilized directly for a range of heating applications. A centralized distribution system is assumed for the synthetic methane gas produced from co-located SNG plants, with the reference case designed to ensure that the industrial clean energy requirements (585,936 GJ) in Makwanpur District are entirely replaced by the generated SNG. The method chosen for hydrogen production involves water electrolysis using PEM electrolyzers. CO_2_ is assumed to be sourced from a nearby Cement Industry named Hetauda Cement, which has an estimated daily cement-making capacity of 750 tonnes when operated at full capacity, resulting the possible CO_2_ emissions of 489.75 tonnes Eq. (2). For this study, it is assumed that the clinker is also produced in the factory to avoid confusion related to the emission factor. The cost of the equipment used in establishing the plant is obtained from supplier quotations and literature (Becker et al., 2019). Parameters such as the stages in the reaction, temperature, pressure, the type of catalyst (Chein and Wang, 2020), and reaction kinetics have been extensively studied and are known to exert a significant influence on the efficiency of the SNG production plant.

$$\text{Emission }E_{\text{CO}2}=\text{Cemen Production }\left(I\right)*\text{Emission Factor}\left(\epsilon \right)$$ (2)

(Lei et al., 2011)

ϵ=0.653

(Lei et al., 2011).

### 2.2 Models

**FIGURE 2:**
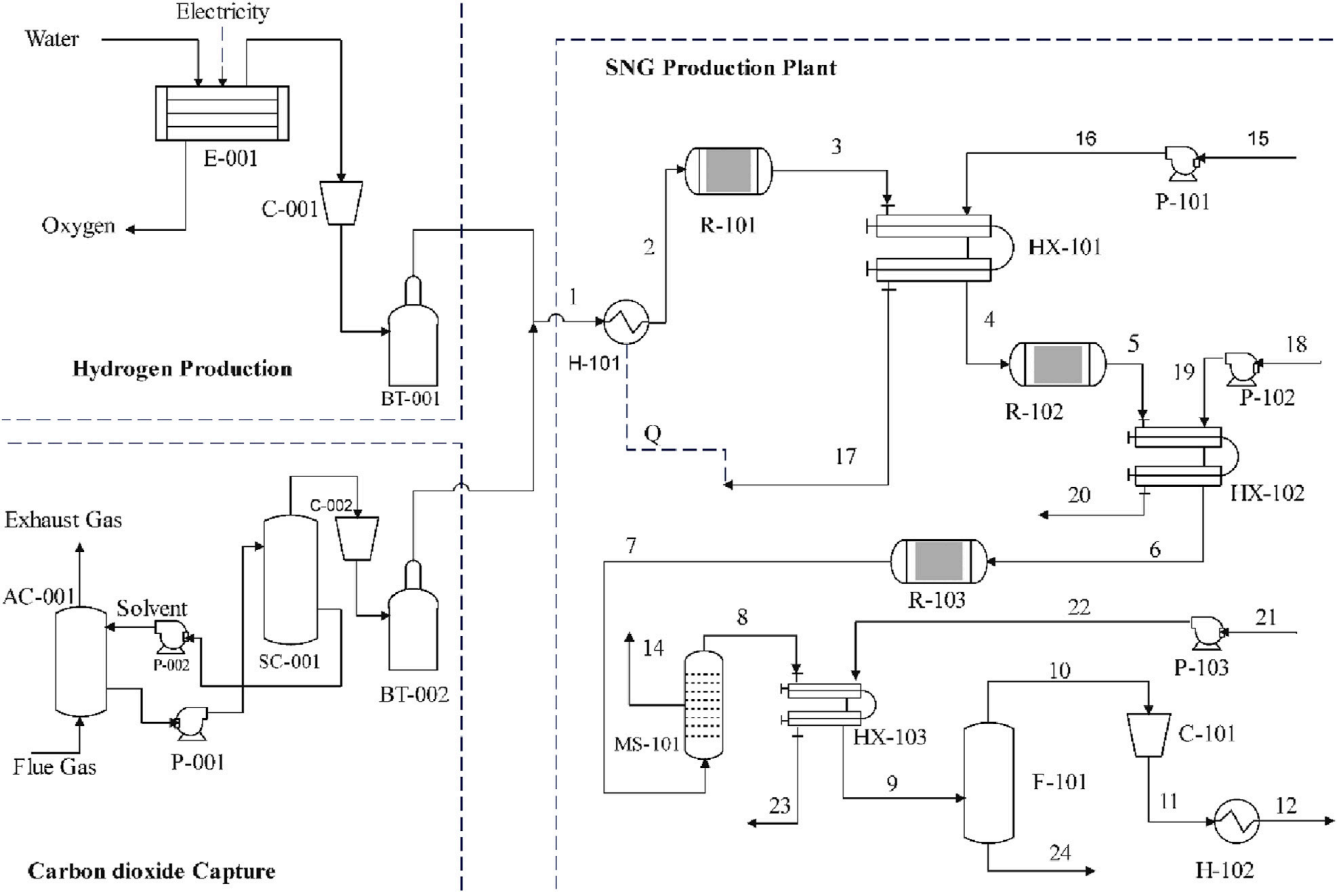
Schematic of the process diagram of the SNG production system.

The process simulation model employed in this study was developed in Aspen Plus software, a process modeling tool designed to replicate real-world chemical processes. The following section depicts the process model developed through the Aspen Plus software as in Figure 2 and the details are tabulated in Table 1.

**TABLE 1:** Equipment code with corresponding equipment name.

Code	Equipment name	Code	Equipment name
E-001	Electrolyzer	H-101	Pre-heater
C-001	Compressor for H_2_	R-101	Packed bed reactor 1
BT-001	Tank for H_2_	HX-101	Heat Exchanger 1
AC-001	Absorption Column for CO_2_	R-102	Packed bed reactor 2
SC-001	Stripping Column for CO_2_	HX-102	Heat Exchanger 2
P-001	Pump for solvent	R-103	Packed bed reactor 3
P-002	Pump for solvent	MS-101	Membrane Separator 1
C-002	Compressor for CO_2_	HX-103	Heat Exchanger 3
BT-002	Tank for CO_2_	F-101	Flash Separator
P-101	Pump for coolant 1	C-101	Compressor for SNG
P-102	Pump for coolant 2	H-102	Cooler
P-103	Pump for coolant 3		

#### 2.2.1 Electrolysis

In the present work, the electrolytic production of hydrogen is envisioned to be geographically co-located with hydropower-generated electricity to meet the demand for green hydrogen in SNG production. The process of water electrolysis involves the breakdown of the water into hydrogen and oxygen by a direct supply of current as shown in the following equation: (Shiva Kumar and Himabindu, 2019):

Anode: ${OH}^{-}\to \frac{1}{2}O_{2}+H_{2}O+2e^{-}$

Cathode: $H_{2}O+2e^{-}\to H_{2}+{2OH}^{-}$

Overall: $H_{2}O+energy\to {2H}_{2}+O_{2}$

The Aspen Plus Software was used to examine a Proton Exchange Membrane (PEM) electrolyzer with a 70% efficiency (Wirkert et al., 2020). Subsequently, the hydrogen generated from the electrolyzer was simulated to be fed into the reactor at 25 bars from a high-pressure buffer tank compressed using a pneumatic reciprocating compressor. The simulations revealed that a 2 TPD SNG plant would require approximately 42 kg/h of hydrogen gas, along with 378 kg of deionized water and 2,520 kW of electrical power. An integrated ∼140 kW air compression unit delivers air at a pressure of 5 bars to operate a pneumatic hydrogen compressor to deliver hydrogen at 25 bars maintaining a 42 kg/h flow rate. Additionally, a valuable by-product of 336 kg/h of oxygen would be concurrently generated. However, it should be noted that the cost of selling oxygen was not considered in this analysis. Figure 2 display the detailed of the major components. The specifications for the electrolyzer were verified through a quotation from the manufacturer “Light Bridge,” based in South Korea.

#### 2.2.2 Amine based carbon dioxide (CO_2_) capture for CO_2_ capture from cement industry

Numerous advanced methods exist for capturing carbon dioxide, including adsorption, physical absorption, chemical absorption, cryogenic separation, and membrane-based absorption. Among these alternatives, amine-based separation technology stands out as a well-established and commercially viable technique. It can be integrated into an existing cement plant in Nepal for extraction of CO_2_ from the flue gas (Bosoaga et al., 2009; Plaza et al., 2020; Devkota et al., 2021). The simplified technical analysis of the CO_2_ capture plant was done in Aspen taking reference from Plaza et al., 2020; Devkota et al., 2021 as in Figure 2, and the optimum size of the equipment and the utilities required for the capture process were obtained. Results from the simulations show that a CO_2_ flow rate of 236.3 kg/h will be required for the daily production of 2 tons of CH_4_ (stored in BT-002 at 25 ℃ ) with an electrical power requirement of 330 kW including the compressor (CO_2_ gas compressor (120 kW), Liquefaction Compressor (90 kW)) and all the Pumps (Cooling water pump (15 kW), Lean MEA pump (15 kW), Rich MEA pump (15 kW), Cooler pump (15 kW), Caustic pump (30 kW) etc. Aqueous monoethanolamine (MEA) was selected as an absorbent for capturing CO_2_ from the flue gas of conventional CO_2_-emitting plants. The Carbon Capture plant efficiency was assumed to be 90%. Detailed major components are presented in Figure 2 and Table 2.

**TABLE 2:** Details of the sized equipment for synthetic natural gas plant.

Specialized unit	Sizing (kW)	Input	Output	Power
				Consumption (kW)
Electrolyzer Unit	2,750	⁃ 378 l/h deionized Water	42 kg/h H2 at 25 bar and 336 kg/h O2	2,659
		⁃ 35,000 l/h Cooling water		
CO_2_ Capture Unit (amine-based)	330	⁃ 12.4% CO_2_ in flue gas at 1.013 bar and 40 ℃	99.9% CO2 at 25 bar and 25 ℃	330
SNG Production Unit	96	⁃ 21.48 kg/h H_2_ at 25 bar and 20.5 bar and 25 ℃	83.33 kg/h of CH_4_ at100 bar and 25 ℃	96
		⁃ 236.25 kg/h CO_2_ at 20 bar and 25 ℃		
Total				3085

#### 2.2.3 Thermo-catalytic Sabatier process to produce Synthetic Natural Gas (CH_4_)

Synthetic Methane (Synthetic Natural Gas) is produced through the Sabatier process, which involves the utilization of CO_2_ captured from industrial flue gas and H_2_ generated via water electrolysis in the presence of a catalyst Eq. (1), Here, the Ruthenium, i.e., 0.5 wt% Ru/γ-Al_2_O_3_ was used for simulations due to its 96% yield to methane gas with no CO production at 300°C (Falbo et al., 2018) whereas Nickel has high selectivity and low cost but is more prone to catalyst deactivation it only gives 80% yield to methane gas along with CO production at 400°C on 20% Ni/γ-Al_2_O_3_ (Chein and Wang, 2020). The SNG production plant was modeled in Aspen Plus software. The reactor used is a packed bed reactor (RPLUG).

The Plant feed of CO_2_ is chosen to be consistent with the 4:1 H_2_:CO_2_ molar ratio from the stoichiometry of the reaction. The CO_2_ is distributed such that the ratio of H_2_:CO_2_ is always greater than 4:1 as suggested by Arita and Iizuka, 2015. The three reactor stages with 80%, 70%, and, 60% CO_2_ conversion efficiency for the first, second, and third stages respectively were selected as stated by Becker et al. (2019) for the following reasons: i) to enable water purging, driving the reverse WGS reaction, ii) to avoid the necessity of bulk recycling with two or fewer reactors, and iii) to enhance reactor efficiency while managing capital costs. The supply of hydrogen and carbon dioxide was set at 42 kg/h at 25 bar pressure and 249.96 kg/h at 25 bar pressure respectively. The electrical power required for the preheaters for the initial heating of the input gases (later the heat from the exothermic reaction is used for preheating using heat exchangers), pumps, and compressor is 96 kW. The kinetics of the CO_2_ methanation on a Ru-based catalyst was used as given by Falbo et al., 2018.

### 2.3 Cost estimation model

In this analysis, the estimation of equipment cost (C) involves a comprehensive consideration of various elements as in Eq. (3). These elements incorporate the cost in the reference year (C_R_), the recommended plant size for the equipment determined using the Aspen Plus model (S), the size in the reference year (S_R_), the chemical engineering plant cost index for the year 2022 (CI_22_), the cost index for chemical engineering plants in the reference year (CI_R_), and a scale factor (sf) that can vary within the range of 0.6–0.8, as depicted in the provided Table 3. (Turton, 2012a; Devkota et al., 2021).

$$C=C_{R}\times \frac{S}{S_{R}}\times \frac{{CI}_{22}}{{CI}_{R}}\times sf$$ (3)

**TABLE 3:** The scale factor of the sized equipment.

Components	Scale factor	References
Hydrogen	0.75	(Green and Perry, 2008; Turton, 2012a; Alnouss et al., 2022)
• Electrolyzer Unit	0.84	
• Reciprocating Compressor		
Carbon Dioxide	0.6	
• Carbon Capture Unit	0.84	
• Compressor		
Synthetic Natural Gas	0.71	
• Heat Exchangers	0.6	
• Reactors	0.8	
• Compressor	0.43	
• Pumps	0.6	
• Cooling tower	0.65	
• Preheater		

Subsequently, the equipment cost derived from the equation above is combined with the expenses for installation, piping, buildings, electrical, and instrumentation costs to compute the total capital investment for each piece of equipment. The annual repair and maintenance cost are accounted for as 2.5% (Ali Khan et al., 2021) of the total capital expenditure, with labor costs being determined using the formula found in R. Turton’s book. The costs for electricity, de-ionized water, and cooling water are taken from the standards set by the Nepal Electricity Authority and various water distribution bodies in Nepal.

The sizing factor for the Electrolyzer and the carbon capture unit was directly taken from literature but the SNG plant uses heat exchangers and cooling towers to reuse the heat and there were lack of similar concept was found in the literature hence the major equipment’s were sized based on the plant schematic in Becker et al., 2019 and sizing factors in Turton, 2012a book.

## 3 Economic assessment

After conducting a technical assessment and equipment sizing for the 2 TPD SNG plant, the cost estimation model was used to calculate the cost of the required equipments. To enhance the accuracy of the cost estimation, the costs were cross-referenced with those provided by suppliers. Selections of NEL-Hydrogen’s “M Series” MC 500 Electrolyzer and BOSCO INDIA were based on technical specifications derived from ASPEN simulation for electrolysis and carbon capture. The total equipment purchase cost, amounting to 44.14 Million NPR (equivalent to 3.42 Million USD), was calculated using Eq. 3 and data from Table 3. This hydrogen production equipment, constituting 62% of the total cost whereas SNG reactor unit and CO_2_ production account for 22% and 16% of the capital expenditure, respectively. The considerable variance in equipment costs is attributed to the notably lower technology readiness level (TRL) of the hydrogen production unit. (Pinsky et al., 2020). The Economic Assessment model was adapted from the economic analysis done by Ghimire et al., 2024.

The annual operating cost encompasses the electricity cost, determined by the average rate offered by the Nepal Electricity Authority (NEA). The computation of feed and cooling water expenses relied on rates provided by Kathmandu Upatyaka Khanepani Limited (KUKL) and data from R. Turton’s book. Estimates for annual maintenance costs for the electrolyzer and other equipment were derived from a study conducted by Ali Khan et al., 2021. The service period for the PEM Electrolyzer was established at 40,000 h. In this analysis the stack exchange cost is not considered in the initial CAPEX and a 19% (Schmidt et al., 2017) of system is added after every 5 years. Insurance, property tax, and labour rates were sourced from the Nepal Government website. (Ministry Of Labour, 2023).

### 3.1 Economy of scale

An economy-of-scale analysis was conducted based on the CAPEX and OPEX data from Table 4 and Table 5 for the 2 TPD plant, along with the sizing factor of various plants and equipment as outlined in Table 3. The reference commercial scale of SNG plant is selected such that to replace the use of conventional fuel (72%) in the Industries of the Makwanpur District as mentioned in Section 2.1.

**TABLE 4:** CAPEX of the SNG production plant.

System	Components/Description (number)	Size	Capital costs		Manufacturer/ Source
		*(Material)*	(130 NPR/USD)		
Electrolyser system	Electrolyser Unit (1)	2.5 MW	**In USD**	**In NPR**	NEL Hydrogen and Jianggsu Minnuo Group Co. Ltd
	(Compact System with the cooling unit, water pumps, Deionizer, Controllers))	*(SS 316)*	1,329,430	171,496,513	
Storage tank	Buffer Storage Tank 1 set (15 pcs)	1000 L at 45bars 5	377,691	48,722,241	Light Bridge Inc
		*(SS 316)*			
Compressor with Cooling Unit	High-pressure compressor with Heat Exchanger (1)	25 to 360 Bar	359,691	48,722,241	Jiangsu Minnuo Group Co. Ltd
Carbon Capture Unit	Reboilers (1), Drivers, Pumps (5), Heat Exchangers (1) etc.)	15%–17% CO_2_ to 99.999% pure CO_2_	476,000	61,404,000	BOSCO India
		*(Mainly SS 304, Stripper Tower and reboiler CS,)*			
SNG Production Unit	Heat Exchanger (6)	at 2.24 m^2^ HE area	219,708	28,342,332	ASPEN PLUS Economic Analyzer and DSB Engineering
	Reactor (3)	*(SS 304)*	327,710	42,2742,590	ASPEN Economics and (Becker et al., 2019)
	Others (1)	Fixed Bed Reactor with 90% Cascaded efficiency of 3 reactors	314,332	40,548,828	
		*(SS 316)*			
		Reciprocating Compressor, Pumps, Cooling Tower, preheaters, etc			
**Additional Costs**		**Unit**		**Cost**	
Total Equipment Costs		154% of the total equipment cost		**USD**	**NPR**
Fixed Capital Investment (FCI) (Purchase equipment installation, Piping, Electrical installation, Building, land, Location factors)		(Towler et al., 2008; Turton, 2012b)		3,422,036	441,442,708
Working Capital Investment 10%		20% of FCI		5,269,936	685,091,707
and Contingency Cost of 10% (Towler et al., 2008)				1,053,987	135,964,354

With no revenue expected during the first operational year, given the construction, establishment, and testing phases, a preliminary liquidity reserve of 10% is included to ensure smooth operations. Furthermore, a contingency cost of 10% is incorporated into the project budget to address unforeseen fluctuations (Authors Estimation).

**TABLE 5:** OPEX of the SNG plant.

System	Unit	Unit cost	Total annual cost (In NPR)	Total annual cost (In USD)	Source
		(In NPR)			
Electricity	3085 kW	8/kWh	190,649,030	1,466,531	Aspen Plus/ NEA
Maintenance	2.5% of CAPEX		13,701,740	105,398	(Ali Khan et al., 2021)
Deionized Water	500 kg/h	0.001USD/kg	569,400	4,380	(Turton, 2012b
Insurance		0.01 (FCI)	6,850,870	52,699	Devkota et al., 2021)
Catalyst		0.0075* (FCI)	5,138,120	39,524	(Becker et al., 2019)
					And Liaoning Haitai Sci-Tech Development Co Ltd
Labor	30 (Turton, 2012b)	750/day	9,931,350	76,395	(Ministry Of Labour, 2023)

**FIGURE 3:**
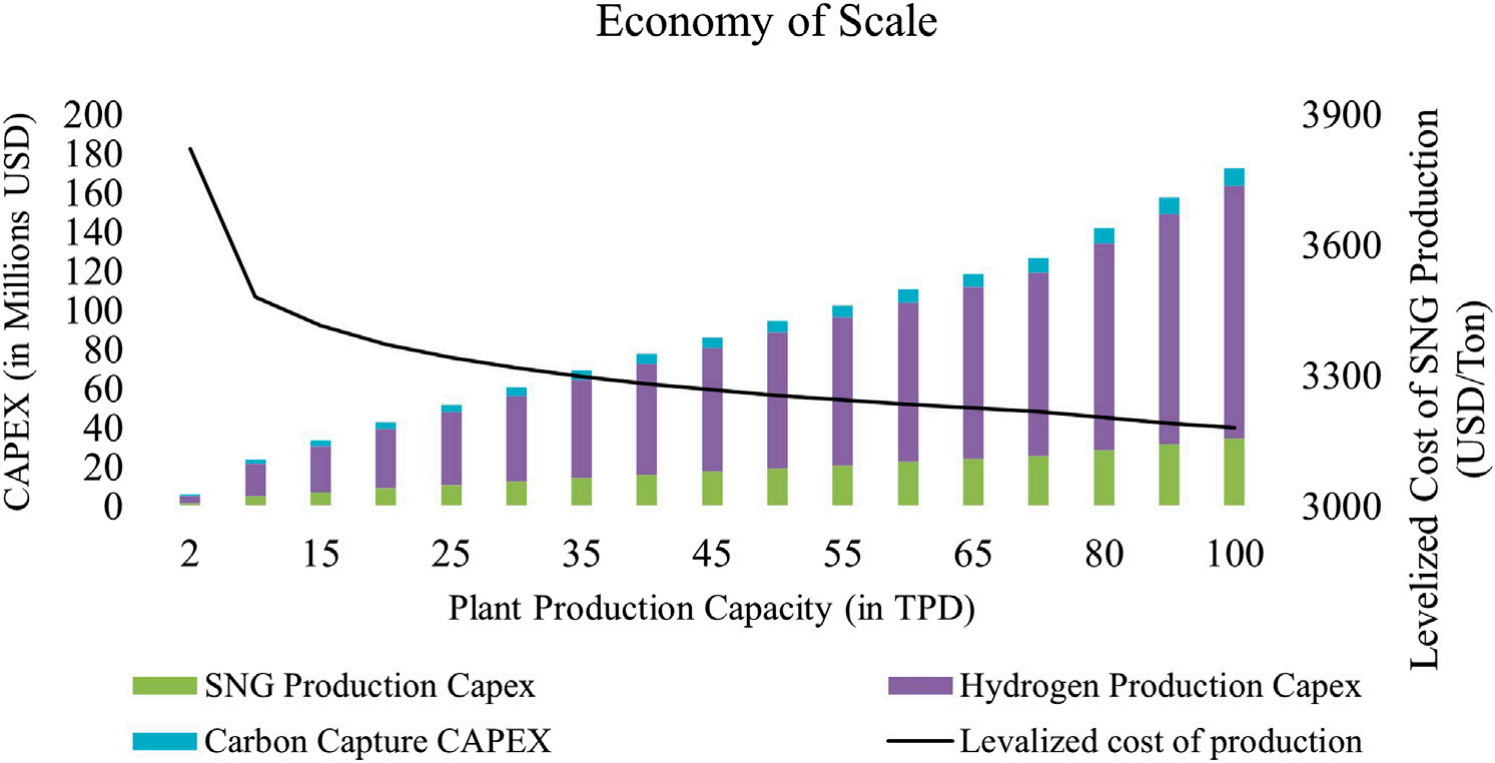
Economy of the scale for SNG production plant in Nepal.

The decrease in the levelized cost of Synthetic Natural Gas (SNG) production is noted as the plant scale is increased as shown in Figure 3. The most suitable size for investment from the demand perspective (equivalent energy required) is determined to be a 40 TPD plant, with an approximate levelized cost of production of 3282.48 USD/ton (423,439 NPR/ton) at an electricity rate of 0.06 USD/kWh (8 NPR/kWh). Beyond this stage, the slope of the levelized cost decreases, but it surpasses the off-takers. However, the slope of the LCOE will not saturate and become more profitable at 100 TPD. In this study, the techno-economic assessment was conducted for both the base scale (2 TPD) and the commercial or reference (40 TPD) to evaluate the feasibility of such plants in Nepal.

### 3.2 Net Present Value

The total CAPEX and OPEX for the 2 TPD and 40 TPD SNG production units were calculated. In both scenarios, annual OPEX exceeds CAPEX. Assuming a selling cost of SNG at USD 3.5 per kg to ensure profit on the cost of production per kg and a positive IRR for both plant scales. The analysis includes cash flow, NPV, and discounted payback period determination. Project acceptability is assessed by comparing the present value of cash inflows to outflows, known as NPV (Turton, 2012b).

**FIGURE 4:**
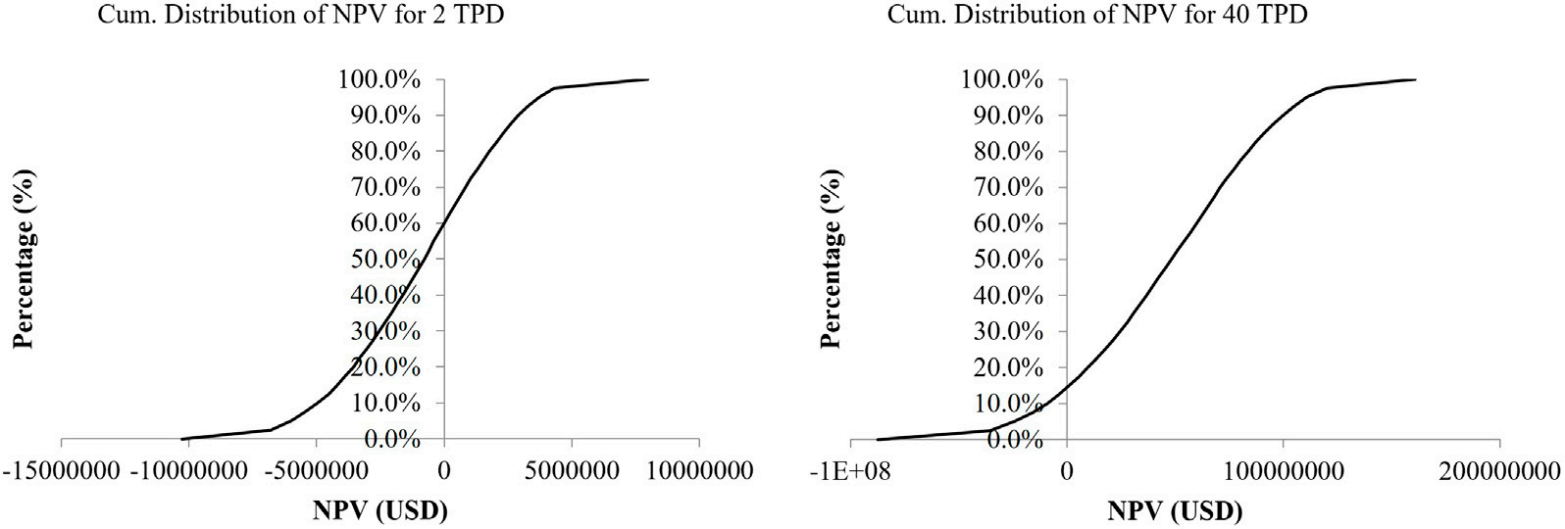
Cumulative distribution of NPV for 2 TPD and 40 TPD SNG Plant (left to right).

Figure 4 Cash Flow Diagram for 40 TPD SNG Plant and 2 TPD SNG Plant with Electrolyzer Stack Exchange

$$\text{PV }\left(i\right)\;I\;\sum_{n=0}^{N}\frac{A_{n}}{{\left(1+i\right)}^{n}}$$

Where, $A_{n}$ = net cash flow at the end of period n i = Discount rate or minimum attractive rate of return (MARR) N = Service life of the project

The IRR was calculated based on a 25-year service life for the SNG production plant system and an 8% discount rate, with corresponding revenues as per the production capabilities (assuming stack change after every 5 years).

For 2 TPD, the NPV comes out to be USD -2 Million (IRR 4% < 8%) suggesting the proposed scale is economically not feasible. For 40 TPD, the NPV comes out to be USD 29 Million suggesting the feasibility of the proposed scale of the plant. The IRR comes out to be 12% for the electricity tariff rate of 8 NPR/kWh (0.061 USD/kWh). The discounted payback time for the 40 TPD plant comes out to be at the end of the 13th year with stack exchange.

### 3.3 Uncertainty and sensitivity analysis

A Monte Carlo simulation was conducted to assess the uncertainty regarding statistical data and the key risk factors associated with investing in the proposed plant. This analysis involves the generation of a wide range of potential outcomes and their associated probabilities through 10,000 simulations. The Monte Carlo simulation was executed using Microsoft Excel software, with the assistance of an optimistic, pessimistic, and most likely values for various cost Parameters as represented in Table 6. Subsequently, the corresponding Net Present Value (NPV) is calculated and plotted to evaluate the range of NPVs and determine the probabilities associated with project-related risks.

**TABLE 6:** Optimistic, pessimistic, and Most Likely Scenarios for various Cost Parameters of SNG Plant.

Cost parameters	Optimistic	Most likely	Pessimistic
CAPEX			
Electrolyzer System	0.6*TCI	TCI	1.6*TCI
Carbon Capture Unit	0.9* TCI	TCI	1.1*TCI
SNG Reactor Unit	0.9* TCI	TCI	1.2*TCI
Other Cost	0.8* (FCI-TEC)	FCI-TEC	1.2*(FCI-TEC)
OPEX			
Maintenance Cost	0.02*FCI	0.025*FCI	0.03*FCI
Electricity Cost	NPR 2/kWh	NPR 8/kWh	NPR 10/kWh
Labor Costs	4% of all other OPEX	5% of all other OPEX	6% of all other OPEX
Deionized Water cost	USD 0.0005/kg	USD 0.001/kg	USD 0.08/kg

In a Monte Carlo simulation for a 2 TPD pilot project, NPV ranged from USD 79.9 Million (IRR: 24.74%) to USD -9.9 Million, with positive NPV in 40% of cases, as depicted in Figure 4. For a 40 TPD commercial scale, NPV ranged from USD 160.7 Million (IRR: 38%) to −87.2 Million, showing positive NPV in over 85% of instances, as illustrated in Figure 4. The positive NPV does not ensure a project profitability but it can be taken as an indicator of the risk of failure. The variance in risk percentage is attributable to differences in profit margins per kilogram of SNG, particularly high for commercial-scale plants.

**FIGURE 5:**
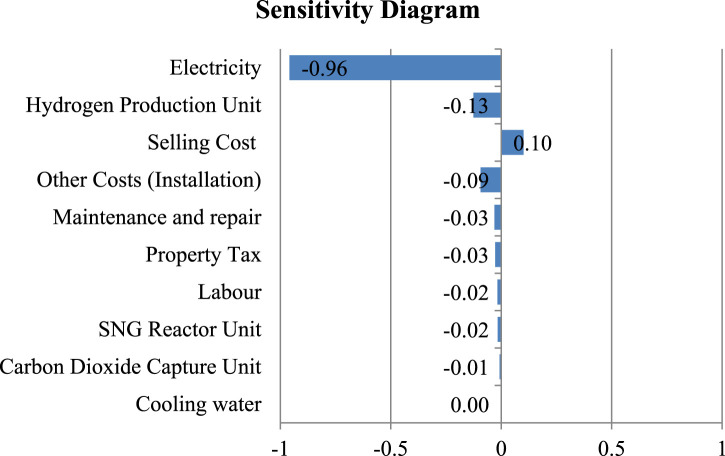
Parameter Sensitivity of SNG Production Plant.

Sensitivity analysis was conducted to identify the most influential parameter affecting NPV. It was found that the most sensitive parameter is the electricity cost followed by electrolyser cost both having negative correlation with NPV as shown in Figure 5. Sensitivity in the Hydrogen production unit is linked to evolving technology and the current low Technology Readiness Level (TRL), leading to cost differences between suppliers and literature. However, the sensitivity of the CO_2_ and SNG unit is low, given minimal discrepancies between literature and market costs for the associated equipment.

**FIGURE 6:**
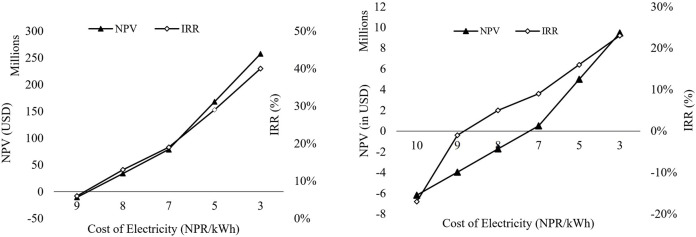
NPV v/s IRR v/s per unit Electricity tariff cost (in NPR) for 40 TPD and 2 TPD plant (left to right)

Considering the results from the uncertainty analysis, which highlights the pivotal role of the electricity tariff rate in the study, sensitivity analysis has been conducted to assess the impact of electricity cost on the Net Present Value and Internal Rate of Return (IRR) of SNG production. The analysis reveals that the levelized cost of SNG production decreases significantly with a reduction in electricity cost, the cost of electricity was taken feasible such that the IRR >8% and NPV is positive at the point. It was found that for the 40 TPD plant to satisfy the condition mentioned above, the electricity cost should be lower or equal to 0.066 USD/kWh (8.7 NPR/kWh). Similarly, for the 2 TPD plant to be profitable (Positive NPV and IRR >8%), the electricity cost must be below 0.053 USD/kWh (6.9 NPR/kWh) at the selling price of 3.5 USD/kg, as shown in Figure 6.

### 3.4 Assessing market competitiveness of Synthetic Natural Gas (SNG) production

Figure 5 illustrates the electricity to be most sensitive parameter for levelized cost of Production (LCOP) and in this analysis different plant scales were evaluated for the price of electricity at which the LCOP of SNG becomes cost competitive to the current Liquified Petroleum Gas (LPG) price. LPG was selected as the point of comparison due to the lack of price competitiveness of SNG production costs when compared to other inexpensive fossil fuels such as coal. Additionally, LPG represents a substantial portion of imported fossil fuel, further justifying its selection for comparison purposes. The highest unsubsidized rate of Liquefied Petroleum Gas (LPG) in Nepal recorded at 227.04 NPR/kg (1.74 USD/kg) (adapted from Bhandari and Pandit, 2018) and this amount has been used for the comparison basis.

**FIGURE 7:**
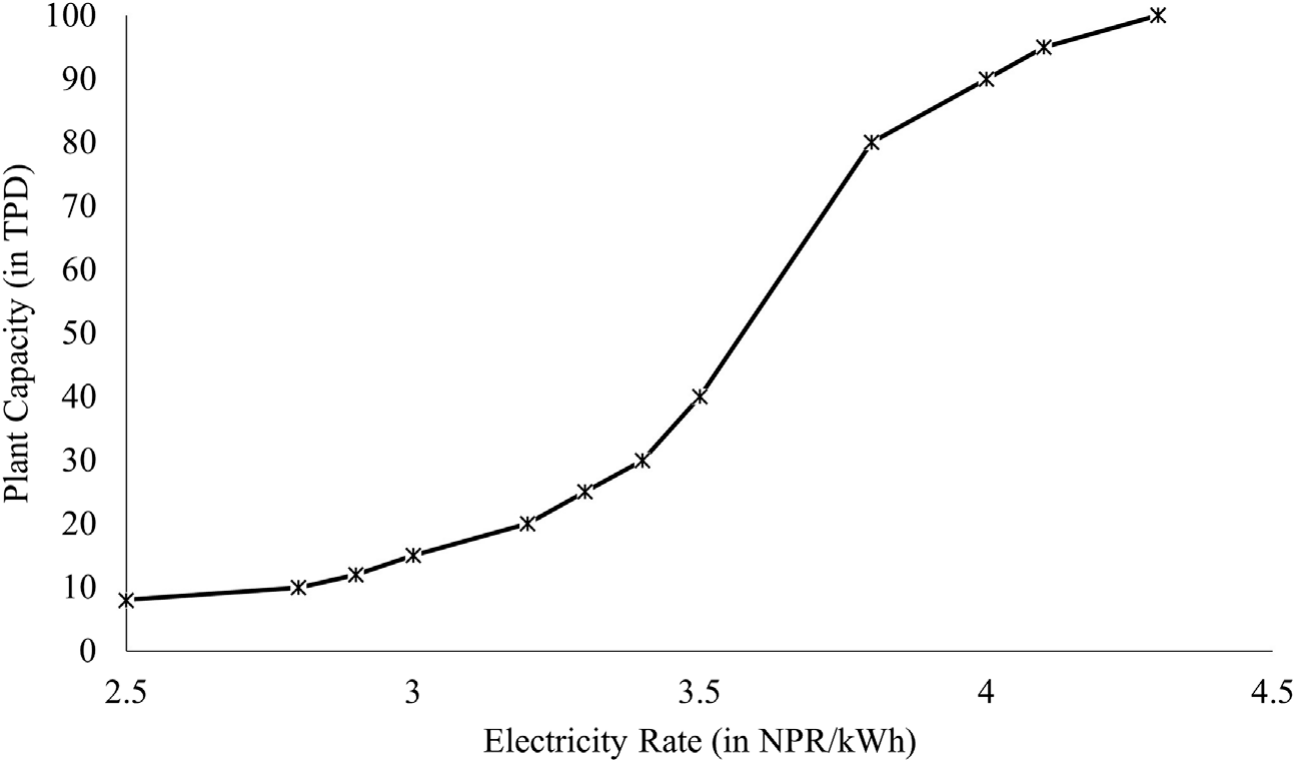
Electricity Rate vs. Feasible Scale of SNG Production Plant (in comparison to LPG prices in Nepal).

At a given plant scale if, $LCOP\;of\;SNG\;\left(energy\;equivalent\;to\;1\;kg\;of\;LPG\right)\leq 227.04\frac{NPR}{\;kg}$ the scale is cost competitive to LPG. The outcomes derived from the graph above demonstrate that, given a subsidized electricity cost of 4.3 NPR/kWh, plant scales equal to or exceeding 100 TPD meet the criteria for comparison and are deemed economically competitive with LPG. Likewise, to ascertain the viability of 40 TPD and 2 TPD plants in terms of market competitiveness against LPG, the subsidized electricity rate must fall below 3.5 NPR/kWh and significantly below 2.5 NPR/kWh, respectively as shown in Figure 7.

## 4 Conclusion

This study involved an economy-of-scale analysis for an SNG production plant, drawing data from various literature and suppliers. The SNG as a fuel can act as a balance between the storage of spilled hydroelectricity from runoff rivers, excessive fossil fuel import, and the economical adaptability of clean fuel in the current heating infrastructure. A techno-economic analysis was conducted for a pilot-scale 2 TPD plant in the Hetauda Industrial district, using technical data from ASPEN Plus. The 40 TPD plant was found feasible to replace the use of CO_2_ emitting fuel with SNG fuel in the Industries of Makwanpur district. The economy of scale analysis was done to find the effect on the levelized cost and CAPEX with increasing plant scale.

Economic analyses for both the 2 TPD and 40 TPD plants were carried out, with CAPEX and OPEX calculated using secondary data from suppliers and a literature review. The levelized cost of production for both the commercial and pilot-scale plants was determined, with a selling cost of 3.55 USD/kg of SNG set to ensure a positive NPV for the 2 TPD plant. Despite higher initial capital requirements for the 40 TPD plants, breakeven was achieved in the 13th year at a discounted rate of 8%. In contrast, the 2 TPD plant found it difficult to breakeven even at the 25th year. The NPV for the 2 and 40 TPD plants were calculated at USD -2 Million with a 4% (< discount rate, 8%) Internal Rate of Return (IRR) and USD 29.2 Millions a 12% (> discount rate, 8%) IRR, respectively.

Furthermore, the Monte Carlo analysis revealed that the 2 TPD plant is about two times riskier than the 40 TPD plant from the investment point of view. Notably, the Monte Carlo equation emphasized a substantial negative correlation between NPV and electricity cost, surpassing correlations with other factors. Consequently, the plants’ sensitivity to electricity rates was simulated, concluding that both scales of plants can become profitable if the electricity cost could be reduced below 6.9 NPR/unit at 3.5 USD/kg selling price. However, for SNG to effectively compete with the LPG market in Nepal, electricity costs should be lowered to 3.5 NPR/kWh for the 40 TPD plant and well lower than 2 NPR/kWh for the 2 TPD plant.

Overall, the viability and scalability of the SNG plant in Nepal depend upon the support from government policies such as promoting affordable electricity for clean fuel application, categorisation of clean fuels as premium fuels, etc. Additionally, the integration of carbon financing mechanisms and use of flue gas heat in the carbon capture can reduce the utility cost and can further enhance the business’s feasibility. It should be noted that this study is constrained by the precision and reliability of simulation software and data obtained from the literature. The study primarily concentrates on SNG production and does not delve into the specifics of auxiliary components. This study is positioned to serve as a stimulus for further research, contributing to policymakers’ and investors’ comprehensive understanding of prospects and enabling well-informed decision-making.

## Data Availability

The original contributions presented in the study are included in the article/Supplementary material, further inquiries can be directed to the corresponding author.
